# Reciprocal Space Mapping Analysis of Mg-Doped InGaN/GaN Heterostructure for Bias-Dependent UV Photodetection Behavior

**DOI:** 10.1021/acsomega.6c04339

**Published:** 2026-08-19

**Authors:** Nor Athirah Mohd Sukri, Momin Shawqi Mohammed Abutawahina, Sha Shiong Ng, Wen-Jih Lin, Victor Colas, Sidi Ould Saad Hamady

**Affiliations:** † Institute of Nano Optoelectronics Research and Technology (INOR), 26689 Universiti Sains Malaysia, Georgetown, Pulau Pinang 11800 USM, Malaysia; ‡ Bruker AXS, New Taipei City 22175, Taiwan, Republic of China; § 137665 Université de Lorraine, CentraleSupélec, LMOPS, Metz F-57000, France

## Abstract

Indium gallium nitride (InGaN) has attracted considerable attention for ultraviolet (UV) optoelectronic applications due to its tunable bandgap and compatibility with GaN-based device platforms. However, the optoelectronic performance of InGaN is strongly influenced by lattice strain, indium distribution, surface morphology, and carrier transport properties. In this work, the UV photoresponse of an Mg-doped InGaN/GaN heterostructure subjected to postgrowth annealing at 600 °C is systematically investigated. Structural properties, including lattice parameters, strain state, degree of relaxation, and indium composition, are examined using high-resolution X-ray diffraction reciprocal-space mapping (HRXRD RSM). The results indicate that the Mg-doped InGaN epilayer is partially relaxed on the GaN layer and exhibits phase separation. The indium composition determined from HRXRD RSM ranges from 4.12% to 8.77%. Atomic force microscopy analysis reveals a step-terrace surface morphology. Optical absorption measurements obtained from ultraviolet–visible spectroscopy show a sharp absorption edge at ∼380–382 nm, corresponding to a bandgap of ∼3.25 eV, indicating suitability for UV photodetection. Hall effect measurements reveal a carrier concentration of ∼10^18^ cm^–3^ and a mobility of ∼188 cm^2^ V^–1^ s^–1^. The fabricated photodetector exhibits a responsivity of 0.024 A W^–1^ and a rise/fall response time of 0.70 s at a bias of 6 V, demonstrating the potential of Mg-doped InGaN for UV photodetector applications.

## Graphical abstract

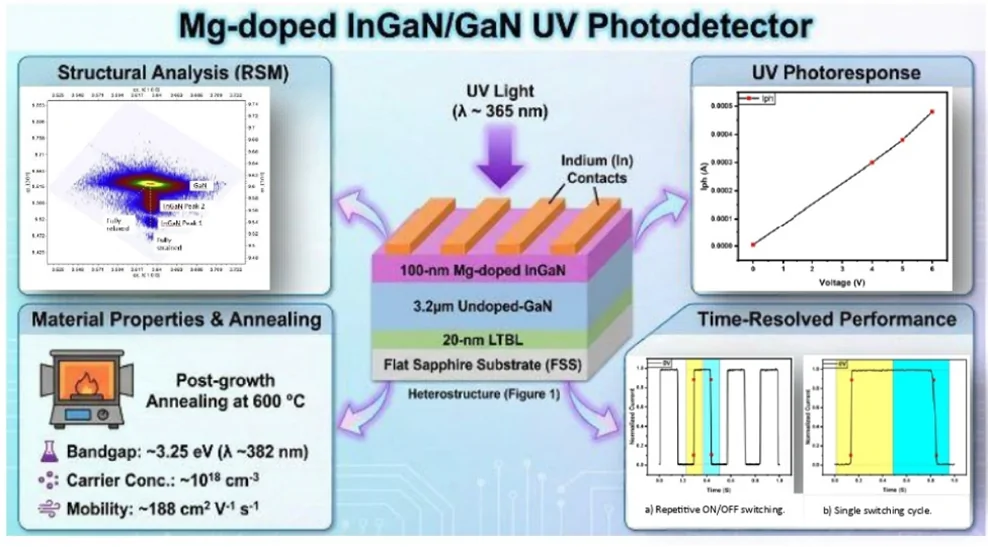

## 1 Introduction

Ultraviolet (UV) optoelectronics have attracted significant attention in recent years due to their applications in photodetectors (PDs), sterilization, sensing, and optical communication systems.^1^ The development of high-performance UV devices critically depends on semiconductor materials with suitable bandgaps, efficient carrier transport, and strong thermal and chemical stability.^2^ Among wide-bandgap semiconductors, GaN is a key material owing to its direct bandgap of ∼3.4 eV, high breakdown field, excellent thermal stability, and radiation resistance.^3–5^

Beyond GaN, InGaN has emerged as a promising alternative due to its composition-dependent bandgap tunability, enabling spectral coverage from the ultraviolet to visible region.^6,7^ However, increasing indium incorporation often introduces significant lattice mismatch with GaN, leading to strain accumulation, defect formation, and compositional inhomogeneity that can degrade optical absorption and carrier transport properties.^8,9^ A further challenge in InGaN-based materials is the realization of stable p-type conductivity. Although Mg is the most widely used acceptor dopant in III-nitrides, its activation in InGaN is hindered by high activation energy, compensation effects, and polarization-induced fields.^10^ Postgrowth annealing is typically required to activate Mg acceptors; however, it may simultaneously influence strain relaxation, surface morphology, and indium distribution in the epilayer.^11,12^ These coupled effects highlight the importance of understanding the relationship between structural, optical, and electrical properties in Mg-doped InGaN systems.

From a characterization perspective, the optoelectronic performance of InGaN/GaN heterostructures is strongly governed by their structural and morphological properties. Strain and relaxation arising from lattice mismatch can modify the band structure, defect density, and carrier-scattering behavior.^13^ HRXRD reciprocal space mapping is widely used to determine lattice parameters, strain state, relaxation degree, and indium composition.^14^ In addition, surface morphology, such as step-terrace structures and step bunching, typically observed in step-flow growth, can influence optical interactions and carrier transport.^15^ AFM provides complementary information on surface roughness and growth evolution.^13^ Optical properties, commonly analyzed using UV–Vis spectroscopy, provide insights into bandgap energy and absorption behavior, which are sensitive to indium composition, strain, and defect states.^1,13^ Electrical transport properties are typically evaluated using Hall measurements to determine carrier concentration, mobility, and resistivity, which are crucial for understanding Mg doping and compositional effects on charge transport.^16^ Overall, UV photodetector performance is determined by the interplay of structural, morphological, optical, and electrical properties.^17^

In recent years, InGaN-based photodetectors have demonstrated strong performance across various device architectures, including Schottky, MSM, p–n, and p–i–n structures.^18,19^ These devices can achieve responsivity ranging from mA W^–1^ to several A W^–1^, along with low dark current, high detectivity, and fast response speed,^20,21^ depending on indium composition, crystal quality, and contact formation. Performance enhancement has been widely achieved through epitaxial growth optimization and improved material uniformity, enabling enhanced photocurrent generation and bias-dependent response behavior,^22^ while some devices even operate in self-powered mode.^23^

Most previous studies have focused on device optimization through heterojunction engineering, contact engineering, and control of growth conditions.^24–26^ In heterojunction approaches, graphene/InGaN structures utilize transferred graphene layers to form Schottky junctions that enhance carrier separation and transport, thereby improving responsivity and response speed.^27,28^ However, interfacial contamination and scalability limitations associated with graphene transfer remain critical challenges for large-area device fabrication. In contact engineering, Ti/Al/Ni/Au multilayer electrodes combined with rapid thermal annealing are commonly employed to form ohmic contacts by reducing barrier height and contact resistance.^26^ For growth control, indium composition is typically tuned by adjusting the MOCVD growth temperature and the TMIn/TMGa precursor ratio, where lower growth temperature or higher TMIn flux promotes indium incorporation in In_*x*_Ga_1–x_N layers.^29^ However, this approach is highly sensitive to strain accumulation and phase separation, often resulting in compositional nonuniformity and defect formation at higher indium content.

In contrast, the present work employs fixed MOCVD growth conditions, yielding an indium composition of ∼18.4%, where unavoidable indium-rich compositional fluctuations, together with Mg-related defect states, may contribute to carrier trapping and modulation of photocarrier lifetime, leading to the observed photoconductive gain. Rather than focusing on bandgap tuning through precise compositional control, this work explores the role of defect- and fluctuation-assisted transport in determining device response. Although strain effects in InGaN have been widely studied, the relationship between strain relaxation observed by RSM and self-powered UV photoresponse remains poorly understood, especially in partially relaxed, compositionally nonuniform InGaN layers. In addition, few studies have correlated RSM-derived strain evolution with UV photodetection performance in Mg-doped InGaN/GaN structures.

## 2 Results and Discussion

The HRXRD RSM approach was employed to evaluate the lattice parameters, strain state, and relaxation behavior of the InGaN layer. RSM patterns were recorded around the asymmetric GaN (105) reflection to probe both in-plane and out-of-plane lattice parameters.^30^

Figure 1 shows the HRXRD RSM patterns measured around an asymmetric reflection of the InGaN/GaN heterostructure. The diffraction intensity is plotted as a function of the in-plane reciprocal lattice vector *Q*_*x*_[100] and the out-of-plane reciprocal lattice vector *Q*_*z*_[001]. The GaN layer exhibits a sharp, high-intensity peak, corresponding to a fully relaxed reference lattice. Two distinct diffraction features from the InGaN layer are also resolved. A small but detectable horizontal shift (*Q*_*x*_) of these InGaN peaks relative to the GaN peak indicates that the InGaN layer is partially relaxed, albeit to a low degree.

**Figure 1:**
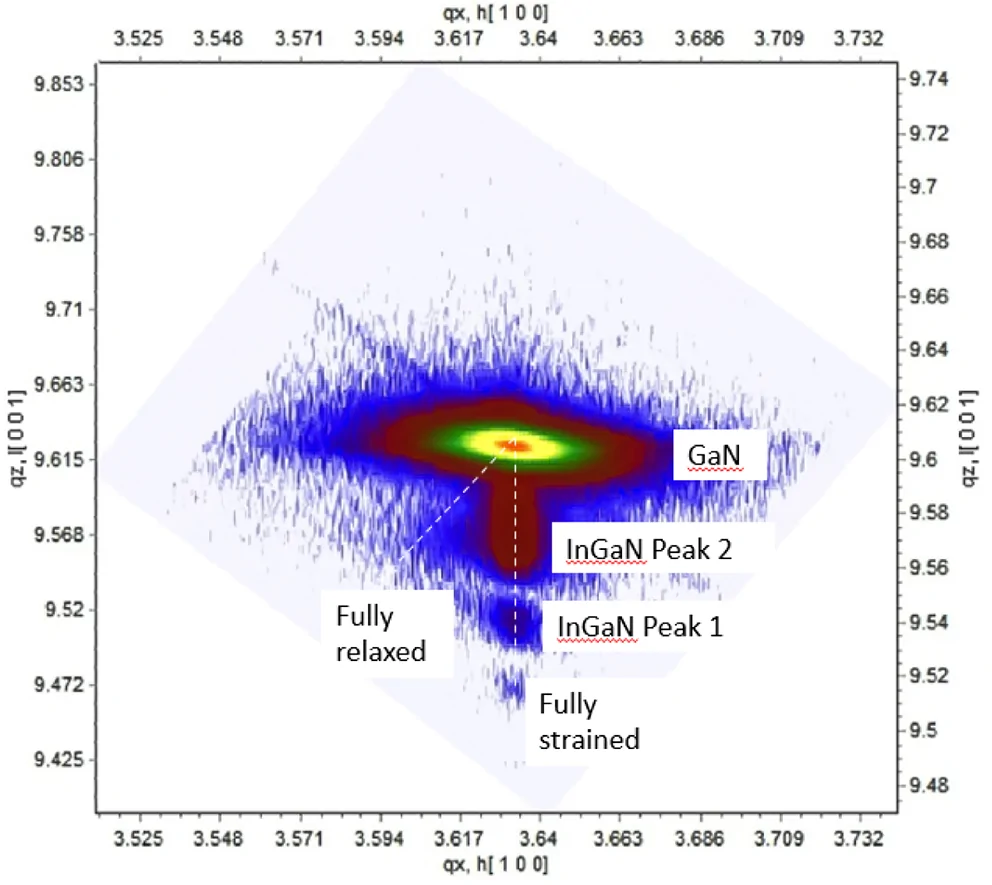
High-resolution X-ray diffraction reciprocal space mapping pattern for Mg-doped InGaN/GaN heterostructure measured around the (105) Bragg peak of GaN.

The observation of phase separation in the InGaN layer is supported by fitting simulations, which reveal multiple indium compositions rather than a single uniform value. This nonuniform indium distribution is further confirmed by RSM analysis, which shows a two-dimensional intensity map with multiple diffraction features associated with the InGaN layer.^31^ In the RSM, the presence of two distinguishable InGaN-related peaks indicates the coexistence of regions with different lattice parameters. By extracting the in-plane and out-of-plane reciprocal lattice vectors (*Q*_*x*_ and *Q*_*z*_), converting them into lattice constants, distinct indium compositions can be identified, confirming that the InGaN layer is not compositionally uniform.

In RSM measurements, the diffraction peak positions are initially reported in terms of detector coordinates (*P_x_*, *P_z_*) rather than in reciprocal lattice units.^32^ As a result, the reciprocal lattice vectors *Q*_*x*_ and *Q*_*z*_ cannot be directly extracted from the raw RSM image. To overcome this limitation, coordinate transformation equations are used to convert the measured peak positions (*P_x_*, *P_z_*) into the corresponding reciprocal-space coordinates (*Q_x_*, *Q_z_*). This approach provides an alternative, more direct method for determining reciprocal lattice parameters without relying on fitting-based simulation models.

Using the known relaxed lattice constant of GaN (*a* = 3.1891 Å) and (*c* = 5.1855 Å),^33^ the theoretical in-plane reciprocal lattice vector *Q*_*x*_,_GaN_, and *Q*_*z,*GaN_ were calculated based on the hexagonal crystal geometry, using eqs 1 and 2,^34^

1 $$Q_{x,GaN}=\frac{2\pi \sqrt{4/3}\times \left(h^{2}+k^{2}\right)}{a_{\mathrm{GaN}}}$$

2 $$Q_{z,GaN}=\frac{2\pi \times l}{c_{\mathrm{GaN}}}$$

where *a*_GaN_ and *c*_GaN_ are the lattice constants of GaN, and *h*, *k*, *l* are the Miller indices of the measured reflection.^35^ In the reciprocal space maps, the diffraction features are displayed as intensity distributions with respect to the experimental position coordinates *P*_*x*_ and *P*_*z*_. These coordinates correspond to detector and geometry-dependent positions and do not directly represent the absolute reciprocal lattice vectors *Q*_*x*_ and *Q*_*z*_. Therefore, a conversion procedure is required to accurately determine the physical reciprocal-space coordinates of the diffraction peaks using eqs 3 and 4.

3 $$Q_{x}=S_{x}P_{x}+Q_{x0}$$

4 $$Q_{z}=S_{z}P_{z}+Q_{z0}$$

Here, *S*_*x*_ and *S*_*z*_ are scaling factors relating the measured momentum transfer to the reciprocal-space coordinates, while *Q*_*x0*_ and *Q*_*z0*_ account for offsets due to the detector geometry or experimental alignment. *P*_*x*_ and *P*_*z*_ represent the projected components of the scattering vector along the horizontal and vertical directions, respectively.^36^ Allowing the conversion of experimental coordinates into absolute reciprocal space values. The reciprocal lattice coordinates of the InGaN layer were subsequently obtained. The extracted in-plane (*a*) and out-of-plane (*c*) lattice parameters were subsequently used to evaluate the in-plane (ϵ_||_) and out-of-plane (ϵ_*⊥*_) strain of the InGaN layer^38^ using eqs 5 and 6, respectively. The strain values were calculated by comparing the measured lattice parameters with the corresponding relaxed and measured lattice constants of InGaN.

5 $$\epsilon_{||}=\frac{\alpha_{measured}-a_{relaxed}}{\alpha_{relaxed}}$$

6 $$\epsilon_{⊥}=\frac{c_{measured}-c_{relaxed}}{c_{relaxed}}$$

where *a*_*measured*_ and *c*_*measured*_ are the lattice parameters obtained from RSM, while *a*_*relaxed*_ and *c*_*relaxed*_ represent the relaxed lattice constants of InGaN. For wurtzite III-nitride materials grown along the *c*-plane sapphire substrate, the in-plane and out-of-plane strain components are related through elastic constants according to biaxial strain theory:^38^

7 $$\varepsilon_{⊥}=-2\frac{C_{13}}{C_{33}}\varepsilon_{||}$$

Equation 7 reflects the Poisson-type elastic response of the crystal, in which the elastic coupling to the in-plane stress determines the lattice deformation along the growth direction. Both C_13_ and C_33_ are the stiffness coefficients of wurtzite InGaN that quantify the interaction between the basal-plane compression (or tension) and the strain along the *c*-axis.^39^ In this work, the elastic constant ratio for GaN *C*_13_ */C*_33_ = 103/405 GPa, InN *C*_13_ */C*_33_ = 92/224 GPa,^40^ consistent with reported values for III-nitride alloys. By substituting eqs 5 and 6 into eq 7, the strain relation can be rewritten directly in terms of lattice parameters. Since InGaN is a ternary alloy, its relaxed lattice constants depend on the indium composition, *x*. The relaxed lattice parameters are expressed using Vegard’s law,^41^ as in eqs 8 and 9, respectively.

8 $$a_{Relaxed}^{InGaN}\left(x\right)=a_{0}^{InN}\left(x\right)+\left(1-x\right)a_{0}^{GaN}$$

9 $$c_{Relaxed}^{InGaN}\left(x\right)=c_{0}^{InN}\left(x\right)+\left(1-x\right)c_{0}^{GaN}$$

By combining strain analysis, elastic theory, and Vegard’s law, the indium composition of each InGaN peak can be independently determined. The coefficients of these equations are $a_{0}^{InN}=0.35383\;nm$ , $c_{0}^{InN}=0.57034\;nm$ $a_{0}^{GaN}=0.31891\;nm$ and $c_{0}^{GaN}=0.51855\;nm$ .^31^ Thus, the relaxation of each peak can be determined by using eq 10.^42^ This method explicitly accounts for strain effects and provides a more physically meaningful estimate of indium content.

10 $$R\left(x\right)=\frac{a\left(L\right)-a_{◦}\left(S\right)}{a_{{}^{\circ}}^{R}\left(L\right)-a_{◦}\left(s\right)}$$

Here, *a*(*L*) is the measured in-plane lattice constant of the InGaN layer, *a*_◦_(*S*) is the relaxed lattice constant of the underlying GaN template (substrate), and $a_{{}^{\circ}}^{R}\left(L\right)$ is the relaxed bulk lattice constant of InGaN with the corresponding indium composition

Table 1 lists the extracted lattice parameters, strain values, indium composition, and relaxation degree for the two InGaN peaks identified in the RSM. The significant differences between Peak 1 and Peak 2 indicate compositional inhomogeneity and varying strain states within the InGaN layer. Although Peak 1 appears less shifted in reciprocal space compared to Peak 2, quantitative analysis reveals that Peak 1 corresponds to a higher indium content (*x* = 8.77%) than Peak 2 (*x* = 4.12%). Indium is compositionally inhomogeneous, forming In-rich and In-poor regions, which broaden the InGaN peaks. This apparent contradiction arises because the peak position in RSM is governed not only by alloy composition^1^ but also by the combined effects of in-plane strain and relaxation.^43^ The broadening is likely dominated by relaxation-induced disorder or defects rather than indium concentration alone. Peak 1 exhibits a larger compressive in-plane strain (−0.94%) and a lower degree of relaxation (≈1.57%), which suppresses the out-of-plane lattice expansion and limits the vertical peak displacement.

**Table 1:** Structural Parameters Extracted from the Analysis of the HRXRD RSM Pattern of Mg-Doped InGaN/GaN Heterostructure

	InGaN Peak 1	InGaN Peak 2
Lattice constant, *a* [Å]	3.1896	3.1898
Lattice constant, *c* [Å]	5.2463	5.2138
In-plane strain, *ϵ*_||_ [%]	–0.94	–0.42
Out-of-plane strain, *ϵ*_⊥_ [%]	+0.29	+0.14
Indium content, *x* [%]	8.77	4.12
Relaxation [%]	+1.57	+4.90

In contrast, Peak 2 shows a smaller compressive strain (−0.42%) but a significantly higher relaxation degree (≈4.90%), allowing its lattice parameters to approach the relaxed InGaN values and resulting in a larger apparent shift in reciprocal space despite its lower indium content.^44^

From the valence force field (VFF) model, total strain energy is dependent on the atomic position in the wurtzite structure and lattice constants.^45^ The presence of foreign atoms, such as Mg, will alter the atomic positions and lattice constants in the wurtzite structure of InGaN thin films. Considering that the atomic radius of Mg of 1.60 Å is greater than Ga of 1.22 Å, the presence of Mg will further increase the total strain energy by introducing more compressive strain to the InGaN thin films^46^ The higher the doping level, the greater the compressive strain applied to the thin films. Instead of getting an indium composition of 18.4% (V/III = 16562) under the undoped InGaN recipe in the previous study, the maximum attainable indium composition was ∼10%, which indicated that all the Mg-doped samples were under high strain

Understanding further the origin of the enhanced photodetector performance, the structural characteristics obtained from XRD analysis were correlated with the carrier transport behavior. The XRD results indicate phase separation into In-rich and In-poor regions, as evidenced by distinct high-In and low-In diffraction peaks. The high-In peak (Peak 1) corresponds to regions with higher indium incorporation but comparatively lower lattice relaxation, while the broader low-In peak (Peak 2) indicates stronger relaxation and compositional inhomogeneity. The incomplete strain relaxation suggests that a significant portion of the strain energy remains elastically stored within the heterostructure,^47^ leading to the possible formation of localized potential fluctuations, based on Figure 2. These fluctuations create spatially varying band edges that act as carrier-confinement or trapping centers within the material system^48^

**Figure 2:**
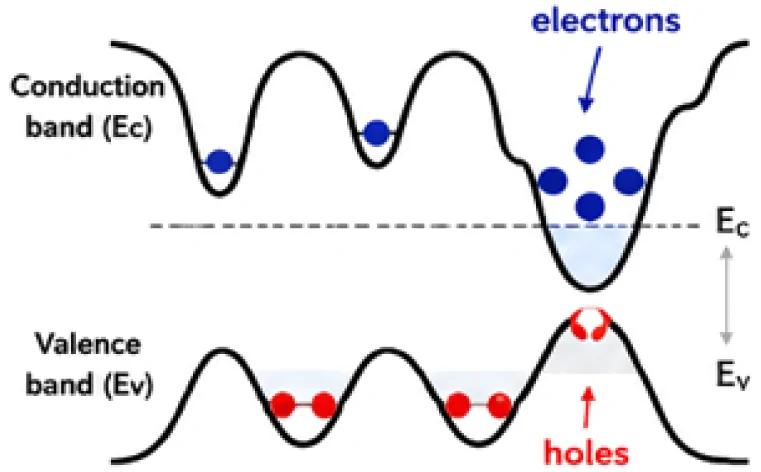
Schematic illustration of carrier transport evolution from delocalized to localized states in the phase-separated Mg-doped InGaN heterostructure.

Although Peak 1 exhibits higher indium content and, consequently, higher strain, this lattice distortion introduces local scattering centers that can degrade carrier mobility. Furthermore, the presence of localized states associated with compositional inhomogeneity can increase the effective carrier lifetime,^49^ as photogenerated carriers may be temporarily trapped in potential minima before recombination.^48^ This trapping effect may suppress the immediate recombination of photogenerated carriers and can contribute to prolonged carrier lifetime.^50^ As a result, the extended carrier lifetime is consistent with an enhanced photoresponse in the photodetector, as a larger fraction of carriers can be collected before recombination, which may support the observed photoresponse under zero-bias operation.

Figure 3 presents the AFM surface topography of the InGaN layer after postgrowth annealing at 600 °C. Figure 2a and b were acquired from two different regions of the sample, using the same 5 μm × 5 μm scan area to evaluate surface morphological uniformity across the film. The average terrace width and step height were determined from multiple terraces across the scanned area. Both images exhibit well-defined terrace-like features and step structures, characteristic of step-flow growth on the c-plane-oriented InGaN surface. Although the overall morphology is similar in both regions, slight variations in terrace arrangement, step distribution, and surface roughness are observed, indicating spatially dependent surface evolution during growth and postgrowth annealing. These variations suggest localized differences in step dynamics rather than significant morphological degradation, consistent with a generally stable epitaxial surface.

**Figure 3:**
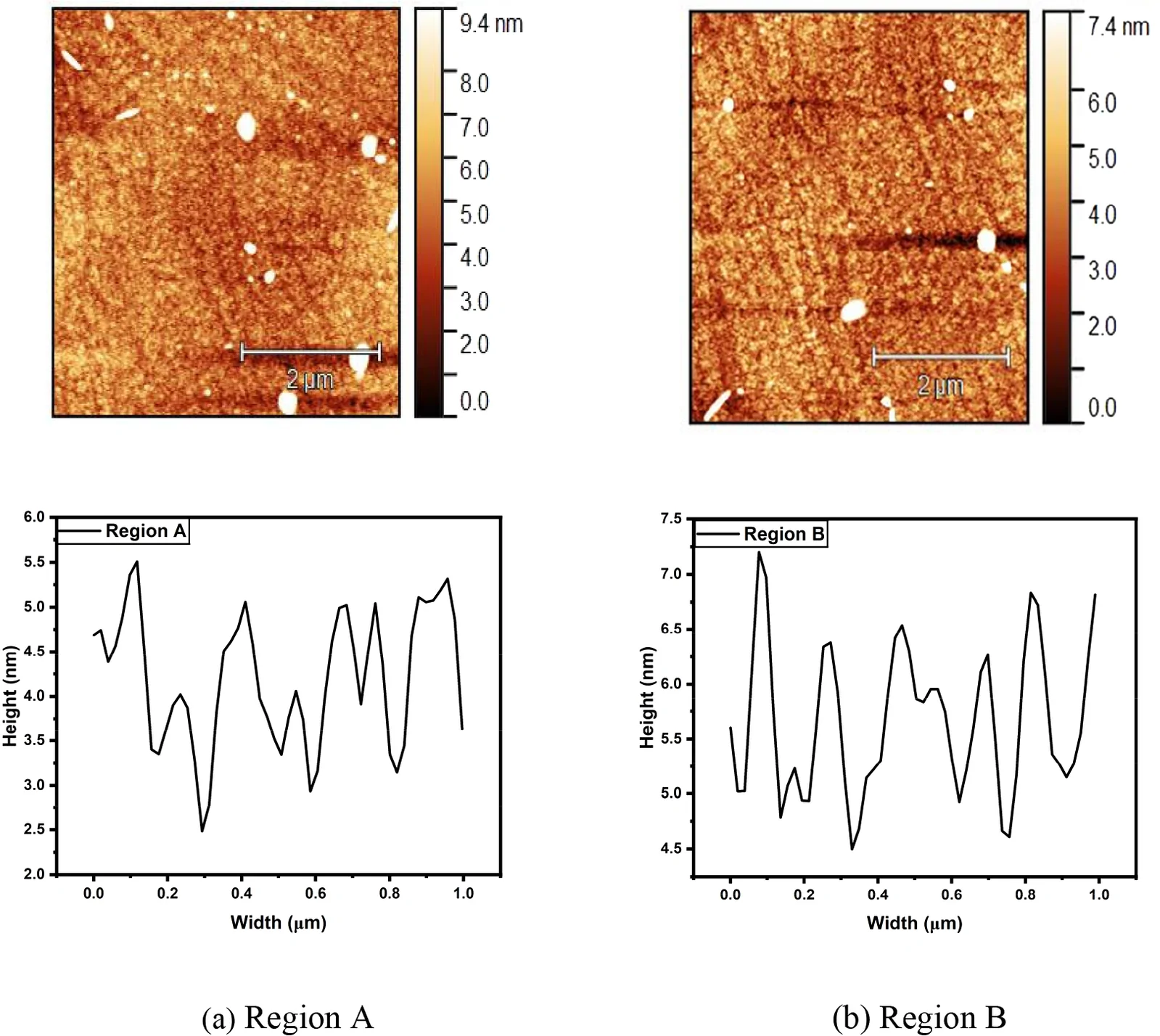
AFM topography images of the Mg-doped InGaN surface measured over scan areas, revealing fine granular morphology and localized surface features. The color scales represent height variations.

The AFM analysis reveals a step-terrace dominated surface morphology across different scanned regions, indicating that the InGaN epilayer grows predominantly via a step-flow mechanism. In Region A, the average step height is 1.33 nm with an RMS roughness of 1.76 nm and an average terrace width of approximately 170 nm. In Region B, the step height increases slightly to 1.41 nm, accompanied by reduced RMS roughness of 1.46 nm and a wider terrace width of approximately 180 nm. The increase in terrace width, together with reduced surface roughness and more clearly defined step terraces, suggests enhanced step ordering and localized step-bunching behavior in certain regions of the film. Although compositional inhomogeneity and phase separation in InGaN are expected due to indium segregation tendencies,^51^ the AFM results indicate that such fluctuations do not lead to the formation of island-like or three-dimensional growth features^52^ at the surface scale investigated. Instead, the surface morphology remains relatively stable and continues to be governed by step-flow growth.

Such step-bunched surface features are commonly associated with the partial relaxation of in-plane strain in lattice-mismatched epitaxial layers.^44^ In this case, the presence of step bunching is consistent with the RSM analysis, which indicates partial relaxation of the in-plane compressive strain through a shift of the InGaN reciprocal lattice point along the *Q*_*x*_ direction. This strain relaxation is accommodated not only elastically within the lattice^53^ but also through surface morphological reorganization. The formation of step terraces and step bunching reflects a balance between minimizing surface energy^54^ and reducing in-plane elastic strain energy.^55^ Additionally, these morphological features are often associated with partial strain relaxation or local variations in indium composition, which can influence the bandgap and absorption edge of InGaN.^56^ This effect improves photon absorption within the InGaN layer.^57,58^

The optical absorption properties of the InGaN thin films were investigated using UV–vis spectroscopy. As shown in Figure 4, the sample annealed at 600 °C exhibits a steep absorption edge at 382 nm, corresponding to the fundamental bandgap transition. The optical bandgap was determined to be 3.25 eV from Tauc plot analysis, where the linear region of the (*αhν*)^2^ versus photon energy (*hν*) plot was extrapolated to intercept the energy axis.

**Figure 4:**
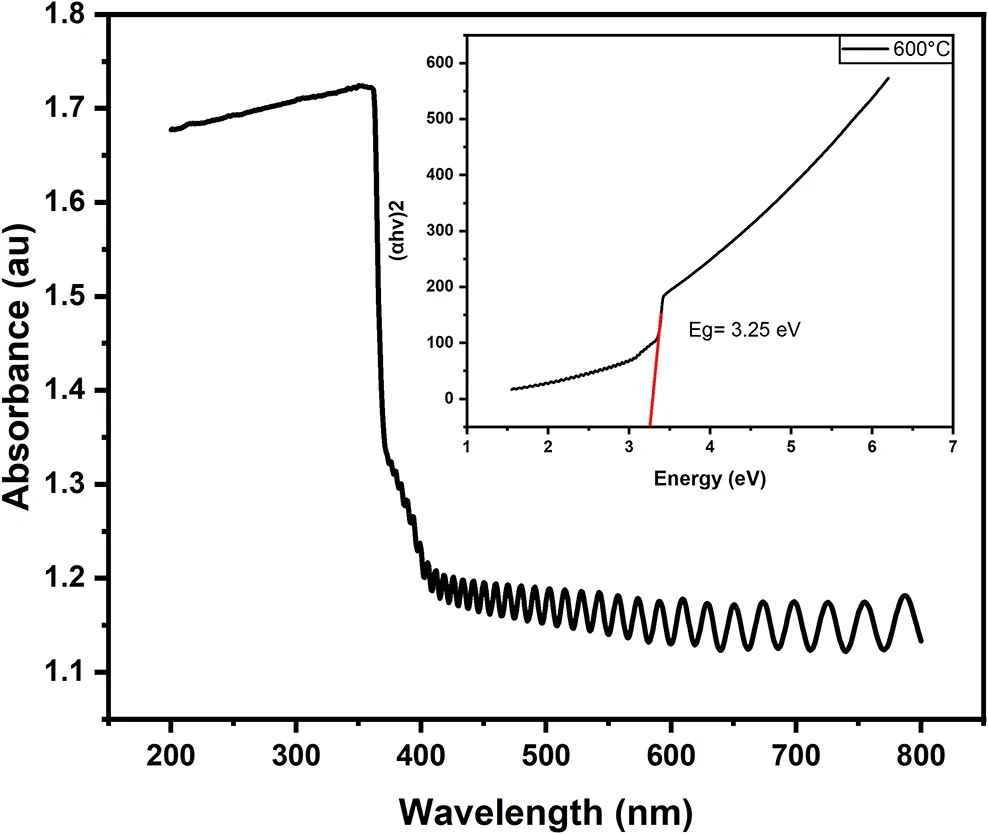
UV–vis absorption spectrum of InGaN annealed at 600 °C, showing a sharp absorption edge at 382 nm (3.25 eV). Inset: Tauc plot ((*αhν*)^2^ vs *hν*) with linear fit used for bandgap determination.

The absorption coefficient (α) was derived from the measured absorbance, and the optical bandgap energy was estimated using the Tauc relation:^59^

11 $${\left(\alpha h\nu \right)}^{2}=A\left(h\nu -E_{g}\right)$$

which applies to the direct bandgap of InGaN semiconductors.

The RSM analysis reveals two distinct InGaN phases with indium compositions of 8.77% and 4.12%, confirming pronounced compositional inhomogeneity. The measured optical band gap of 3.25 eV from UV–vis spectroscopy corresponds closely to the expected value for the phase containing 4.12% indium. This suggests that the lower-indium phase forms the dominant percolating matrix, governing the absorption onset while simultaneously providing a continuous carrier transport network throughout the film. In contrast, the higher-indium phase, though structurally evident, does not govern the fundamental optical edge, likely because it is embedded or clustered within the film.^60^ A bandgap of 3.25 eV fundamentally limits photodetection to wavelengths below 382 nm, conferring inherent UV selectivity as photons beyond this cutoff lack sufficient energy for band-to-band absorption.^61^

The electrical transport properties of the InGaN layer were examined using Hall-effect measurements in a van der Pauw configuration at room temperature. The measured carrier mobility reaches 188.19 cm^2^ V^–1^ s^–1^, accompanied by a carrier concentration of 6.34 × 10^18^ cm^–3^ and a resistivity of 0.00572 Ω·cm. The Hall-effect parameters obtained in this work are comparable to those previously reported for InGaN layers. As summarized in Table 2, the electron mobility of 188.19 cm^2^ V^–1^ s^–1^ exceeds values reported for as-grown InGaN epilayers^64^ and InGaN thick films with comparable indium content,^62^ indicating a substantial reduction in transport-limiting scattering mechanisms following thermal annealing. Although Mg-doped InGaN with unintentional n-type electrical transport typically exhibits higher carrier concentrations, their mobility is significantly lower due to strong ionized impurity scattering and the larger effective mass of holes. In contrast, the present sample maintains a relatively high carrier concentration (∼6.3 × 10^18^ cm^–3^) while preserving high mobility, resulting in low resistivity and efficient electronic transport.

**Table 2:** Carrier Mobility, Conductivity, Resistivity, and Carrier Concentration of the Mg-Doped InGaN Layer Compared with Literature Values for Thick InGaN Films and P-Type or As-Grown InGaN Layers

Sample description	Mobility [cm^2^/V·s]	Conductivity [1/Ohm-cm]	Resistivity [Ohm-cm]	Carrier concentration [cm^–3^]	Refs
Mg-doped InGaN	188.19	191.34	0.0057	6.3429 × 10^18^	This work
InGaN thick films, 5% In	∼148	-	-	∼1 × 10^17^	^63^
p-Type InGaN layers	3 ∼ 16	-	-	6 × 10^18^∼3 × 10^19^	^64^
As-grown InGaN	174	2.734	-	9.8 × 10^16^	^65^

Beyond the absolute values of mobility and carrier concentration, the Hall-effect results provide insight into the electron-dominant transport mechanisms in the annealed InGaN layer. The combination of high mobility and moderate carrier concentration indicates that carrier transport is not limited by ionized impurity scattering but is instead governed by reduced defect- and strain-related scattering, consistent with effective defect passivation during thermal annealing.^65,66^ The observed n-type conductivity also has important implications for the electrical configuration of the heterostructure. Because the Mg-doped InGaN layer exhibits n-type conductivity, the intended p-InGaN/n-GaN junction effectively becomes an n-InGaN/n-GaN isotype heterojunction. Although this differs from the original p–n junction design, the resulting electrical configuration provides distinct advantages that preserve the device’s UV photodetection capability. The coexistence of a relatively relaxed low-In matrix and strained high-In clusters is hypothesized to influence carrier transport within the InGaN layer. The low-In matrix is associated with relatively favorable carrier transport pathways, whereas the high-In clusters introduce localized potential fluctuations that are consistent with carrier localization and trapping effects. These localized states can prolong carrier lifetime by suppressing recombination, thereby facilitating photoconductive gain and enhancing the photoresponse of the n-InGaN/n-GaN heterostructure under UV illumination.

Although Mg is introduced as an acceptor dopant, the Hall measurements indicate electron-dominated transport, suggesting that effective p-type activation was not achieved. This behavior can be understood in terms of acceptor–donor compensation mechanisms commonly reported in Mg-doped InGaN.^67^ In particular, the absence of p-type conductivity after annealing indicates the compensation between Mg acceptors (Mg_Ga/In_) and compensating donor-like defects, while the observed increase in electron concentration suggests the presence of dominant donor species in the thin film. Two widely accepted mechanisms explain this behavior: (a) compensation via nitrogen vacancies (V_N_) acting as donors in the (Mg_Ga/In_ – V_N_) complex, and (b) carbon-related donor impurities (C_Ga/In_) forming (Mg_Ga/In_ – V_N_) complexes that further neutralize acceptor activity.^68–70^ The first mechanism is typically associated with nitrogen-vacancy formation under conditions of high growth temperature or insufficient nitrogen incorporation, where nitrogen atoms are more likely to leave the lattice, thereby generating donor-like defects.^68^ As a result, even after annealing at 600 °C, the incomplete activation of Mg acceptors combined with defect-induced donor compensation prevents the formation of free holes in the valence band (Ev),^71^ as illustrated in Figure 5.

**Figure 5:**
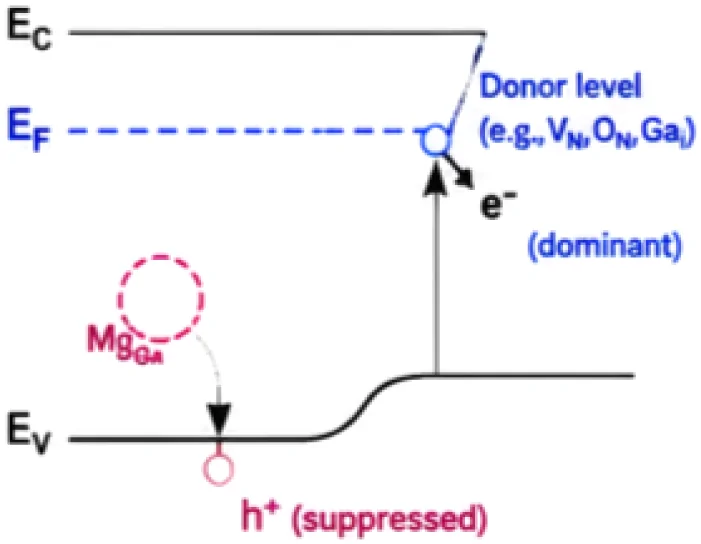
Band diagram of Mg acceptor passivation at 600 °C and donor dominated transport.

Consequently, Mg acceptor levels remain electrically inactive, resulting in negligible hole concentration,^72^ while the Fermi level stays positioned closer to the conduction band (E_c_), consistent with n-type conductivity rather than shifting toward the valence band (E_v_) as expected for effective p-type behavior.^73^ Simultaneously, residual native donor defects such as nitrogen vacancies and unintentional oxygen impurities remain electrically active and contribute electrons to the conduction band.^67^ In the absence of sufficient activated acceptors, these donor states dominate the carrier population, leading to electron-dominated transport. This behavior arises from incomplete Mg activation due to insufficient hydrogen removal and acceptor passivation,^74^ which pins the Fermi level closer to E_c_. Consequently, the device behaves more like an isotype n-InGaN/n-GaN transport system, in which photogenerated electrons dominate the photocurrent due to their higher mobility and longer diffusion length than holes.

Under equilibrium conditions without UV illumination, a measurable dark current is observed in the Mg-doped InGaN/undoped GaN device, indicating residual n-type conductivity despite Mg incorporation. This behavior is attributed to incomplete Mg acceptor activation, compensation by native donor-like defects, hydrogen passivation, and unintentional impurities introduced during growth.^67^ Under dark conditions, thermally generated electrons and intrinsic donor states are proposed to dominate charge transport within the Mg-doped InGaN layer,^75^ enabling carrier motion between the laterally positioned indium-dot contacts under applied bias, facilitated by the approximately ohmic nature of the indium contacts.^26^

The Mg-doped InGaN/GaN heterointerface is expected to induces band bending and an internal electric field due to Fermi-level alignment;^76^ however, in the absence of illumination, no significant photogenerated electron–hole pairs are present,^77^ and the current is primarily influenced by thermally activated carriers and defect-assisted conduction pathways,^67^ as illustrated in Figure 6a. Defect-related scattering centers and deep-level trap states further influence transport by temporarily capturing carriers and reducing mobility.^78^ Upon 365 nm UV illumination, photogenerated carriers fill these trap states, leading to trap saturation, reduced scattering, and increased free carrier density,^79^ as illustrated in Figure 6b. Consequently, carrier lifetime is extended due to suppressed trapping and recombination, enhancing carrier transport between the indium-dot contacts under applied bias.

**Figure 6:**
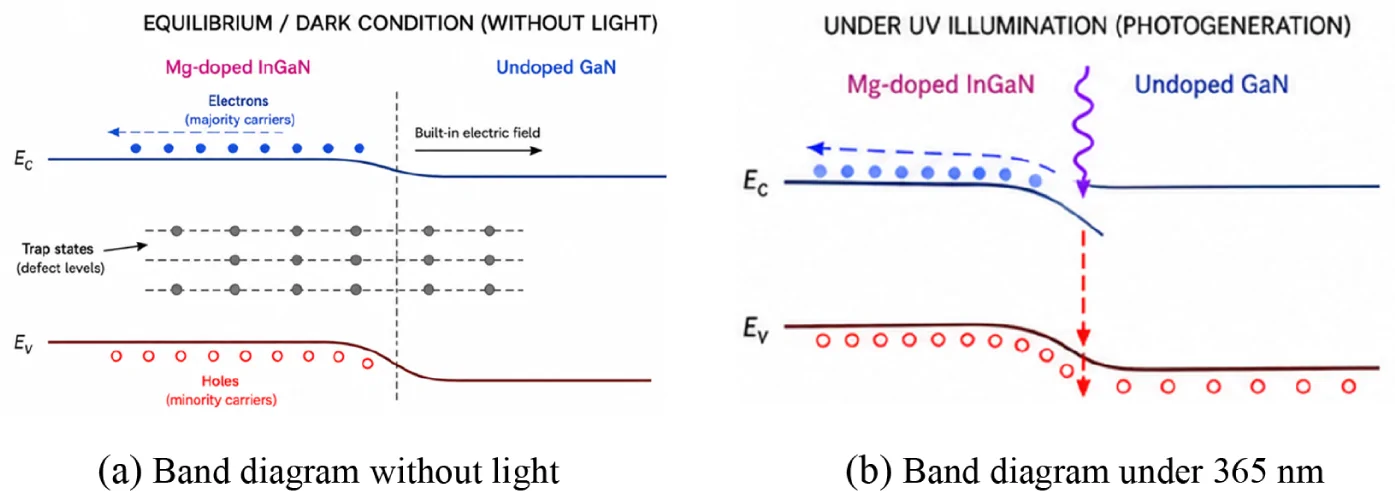
Band diagram of Mg-doped InGaN/GaN photodetectors under (a) dark condition (without light) and (b) UV illumination of 365 nm.

Figure 7 shows the I–V behavior of the Mg-doped InGaN/GaN heterostructure under a −6 to 6 V sweep bias in dark conditions and under UV illumination of 365 nm. The I–V curves in Figure 6a confirmed the ohmic contact behavior, and clear photocurrent responses were observed under UV illumination. As expected, the current under UV illumination increases with forward bias voltage,^80^ which can be attributed to enhanced photogenerated transport across the ohmic contacts.^81^ The results demonstrate that the forward current under UV illumination is significantly higher than that measured in the dark.^82^ The presence of photocurrent at zero bias arises from the internal electric field within the InGaN layer in Figure 6b, which separates photogenerated electron–hole pairs even without an external voltage. This field can originate from built-in polarization effects, band bending at interfaces, or residual strain-induced potentials in the InGaN/GaN heterostructure.^83^ The magnitude of the zero-bias photocurrent reflects the overall photoresponse of the device under illumination, which is a key metric for self-powered UV photodetectors.^84^

**Figure 7:**
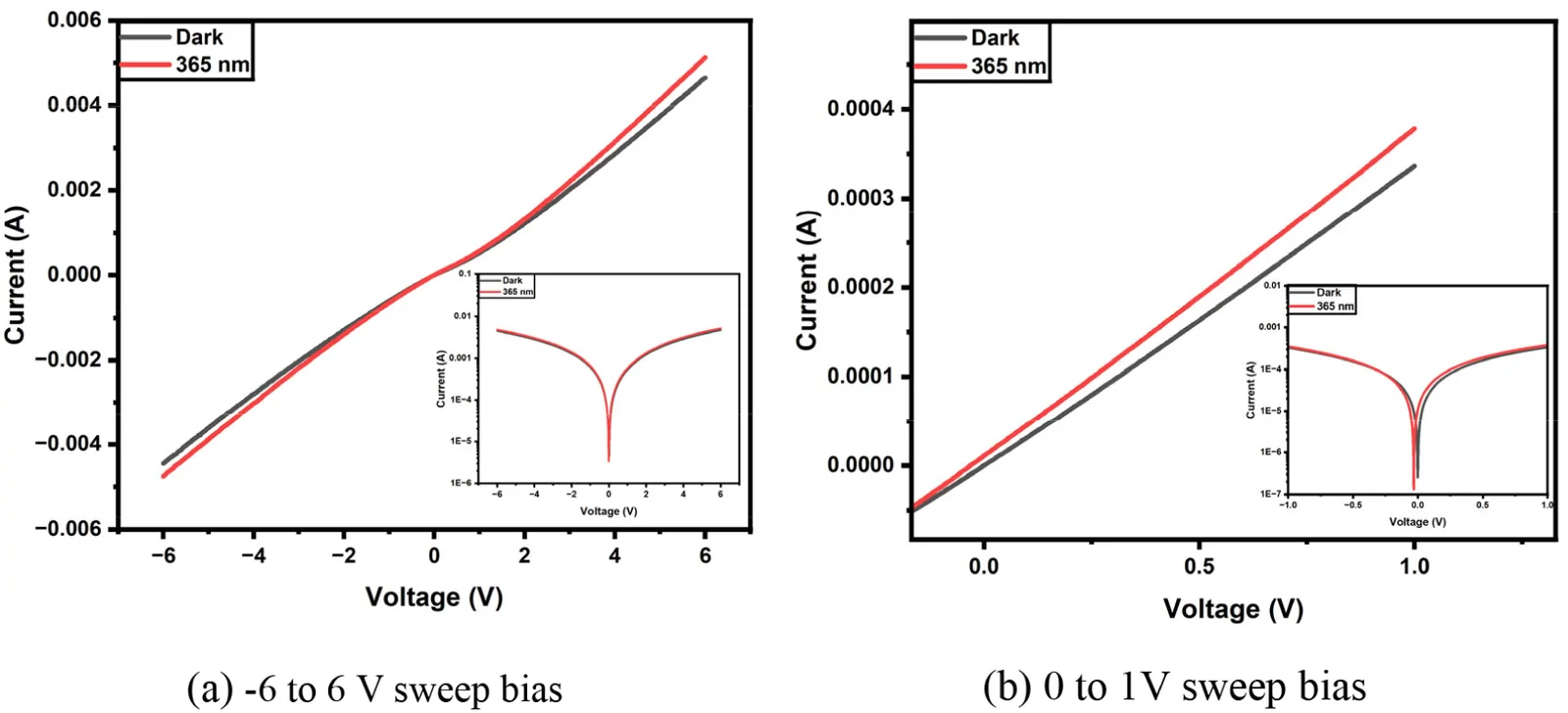
I–V curves of Mg-doped InGaN/GaN photodetectors measured under dark conditions and UV illumination of 365 nm.

The nearly ohmic behavior observed in the I–V characteristics indicates efficient carrier injection and extraction at the metal/InGaN interface. In this work, such contact behavior is advantageous because it minimizes the influence of interfacial barrier effects,^85^ allowing the measured electrical and photoresponse properties to reflect the intrinsic transport characteristics of the InGaN layer primarily.

The UV detection ability of the Mg-doped InGaN was characterized from their electrical response in the presence of UV light illumination.^86^ The net photocurrent (I_ph_) was determined by the formula I_ph_ = I_light_ – I_d_. Here, I_light_ and I_d_ refer to the current under UV irradiation and in the dark, respectively.^85^ At 0 V bias, a measurable photocurrent of 4.52 × 10^–6^ A is observed under an illumination power density of 0.02 W cm^–2^ indicating a net photocurrent generation under zero external bias, which may arise from internal asymmetry in carrier generation, transport, and recombination processes within the Mg-doped InGaN layer. This behavior is likely associated with localized defect states, alloy disorder, and carrier trapping effects, which are expected to influence photogenerated carrier dynamics. Consequently, the device demonstrates the capability for self-powered photodetection.

The linear increase in current with applied bias under illumination, as shown in Figure 8, further confirms that the photocurrent is photogenerated and not dominated by dark leakage.^86^ This behavior demonstrates that, although the sample is not fabricated into a full device with electrodes for optimized extraction, it inherently exhibits self-powered photoresponse due to its internal electric field.^87^

**Figure 8:**
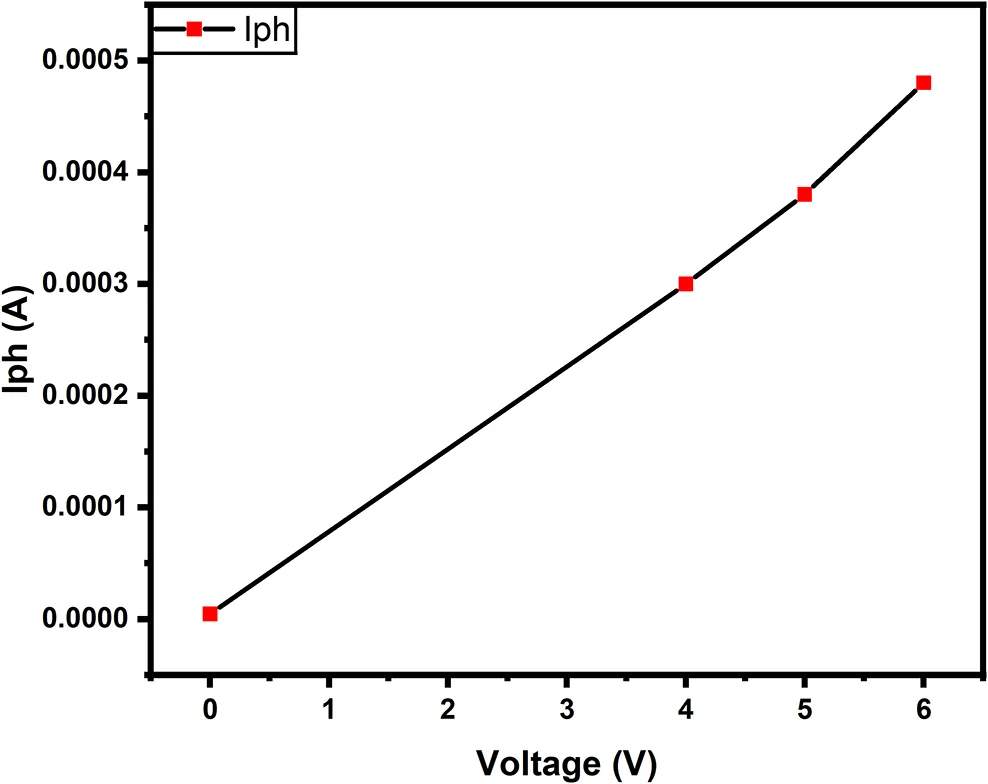
Photocurrent (I_ph_) of the Mg-doped InGaN/GaN UV PD as a function of applied bias voltage.

To further quantify the UV photodetection performance of the Mg-doped InGaN device, key performance figures including responsivity (*R*),^88^ photoconductive gain (*G*), specific detectivity (D*),^89^ and noise-equivalent power (*NEP*)^90^ were evaluated as a function of applied bias voltage. As shown in Table 3, responsivity increases markedly with applied bias, rising from 0.266 mA W^–1^ (at 0 V) to 24 mA W^–1^ (at 6 V), indicating a pronounced bias-dependent photoresponse. This improvement is consistent with the bias-assisted drift transport of photogenerated carriers, which suppresses recombination losses and increases the effective photocurrent.^83^ Furthermore, the specific detectivity (D*) evaluated at a bandwidth of 1 Hz increases from 1.99 × 10^8^ to 6.56 × 10^8^ Jones as the bias is raised from 4 to 6 V. The improved signal-to-noise ratio consequently lowers the NEP from 4.77 × 10^–9^ to 1.45 × 10^–9^ W/cm^2^ Hz^1/2^ and leads to higher detectivity. This improvement indicates enhanced signal-to-noise performance at higher electric fields, primarily due to increased photocurrent relative to dark current noise. The observed trend is consistent with bias-assisted carrier separation, which improves collection efficiency while maintaining relatively stable noise characteristics.

**Table 3:** UV Photodetector Performance of the Mg-Doped InGaN/GaN Device at Various Bias Voltages

Sample description	Voltage [V]	*λ* [nm]	τ_r_ /τ_d_ [ms]	*R* [mA W^–1^]	*G*	D*[Jones] × 10^8^	NEP × 10^–9^	Refs
InGaN	0 V	365	0.002/0.038	0.266	2.01	1.99	4.77	This work
	4 V		0.51/0.69	15.0	1.10	5.05	1.88	
	5 V		0.33/0.46	19.0	1.10	5.77	1.64	
	6 V		0.32/0.37	24.0	1.10	6.56	1.45	
CdS/GaN^a^	0 V	365	0.63/0.77	2.4	-	1.34 × 10^2^		^91^
GaN Nanostructures	3 V	325	332/995	25.9	-	2.83	0.47	^92^
In_0.20_Ga_0.80_N MSM^b^	0.5	360	8.99/9.36	112		6.32 × 10^2^	-	^26^
In_0.15_Ga_0.85_N-MSM	5 V	420	-	8.5	-	-	-	^93^
In_0.14_Ga_0.86_N/GaN MQWs^c^	6 V	365	-	277	-	-	-	^94^
InGaN/GaN MQW	2.5	405	0.02/0.03	25.7	-	4.33	-	^95^
In_0.16_Ga_0.84_N/GaN MQW	–5 V	450	4.6/5.0 × 10^–6^	120	-	-	-	^96^
n-InGaN layer/p-Cu_2_O^d^	0 V	405	8.0/5.0	0.8	-	49.0	-	^97^
InGaN NW PEC^e^	0 V	532	38.5/30.3	0.0215	-	3.46 × 10^5^	-	^98^
PhotodiodesGraphene/ZnO/Silicon Nanowire^f^	–0.5 V	480	1.48/1.27 × 10^–6^	280	-	4.2 × 10^4^	2.59 × 10^–9^	^99^

a CdS/GaN heterojunction (HJ).

b MSM—metal-semiconductor-metal (MSM).

c MQW—multiple-quantum-well.

d InGaN heterojunction (HJ).

e Photoelectrochemical (PEC).

f Photodiode (PD).

The enhancement in responsivity with increasing bias is is consistent with improved field-assisted carrier transport under a stronger electric field, which may reduce carrier transit time and enhance carrier collection before recombination,^100^ while the carrier lifetime remains nearly unchanged, resulting in a relatively stable photoconductive gain from 4 to 6 V. However, zero bias (0 V) has a higher photoconductive gain than 4, 5, and 6 V. This behavior is likely due to carrier trapping in the InGaN layer. At zero bias, photogenerated carriers may be temporarily trapped by defect states or localized states in the InGaN layer, leading to a prolonged effective carrier lifetime and enhanced internal gain. The trap-assisted carrier confinement prolongs the carrier lifetime (τ_life_). If the electron is the majority carrier, then the gain^101^ can be written as in eq 12

12 $$G=\frac{\tau }{\tau_{T}}$$

where τ is the carrier lifetime and τ_1_ is the carrier transit time. During illumination, photogenerated electrons drift through the device within an average transit time τ_1_. When the carrier lifetime exceeds the transit time (τ ≥ τ_1_),^86^ electrons can reach the anode before recombination occurs. To preserve charge neutrality, additional electrons are continuously injected from the cathode and repeatedly transported across the photoconductor until recombination takes place. Consequently, a single photogenerated carrier can contribute to multiple charge transport cycles, resulting in a photoconductive gain greater than unity.^102,103^ In the present device, trap-assisted processes are proposed to prolong the carrier lifetime, resulting in a situation where the carrier lifetime exceeds the transit time (τ > τ_1_). This condition could explain the observed enhancement in photoconductive gain, particularly under zero-bias operation. For a photoconductor, the photocurrent can be expressed as a function of the photogeneration rate and the photoconductive gain^104^

13 $$I_{ph}=gALe.G$$

where *g* is the generation rate of excess carriers (cm^–3^ s^–1^), *L* is the channel length, *A* is the cross-sectional area, *q* is the elementary charge, and *G* is the photoconductive gain. Since responsivity is defined, $R=\frac{I_{ph}}{PA}$ , substituting *I_ph_* = *gALe*.*G*

14 $$R=\frac{gLq.G}{P}$$

This relationship means that responsivity increases as photoconductive gain increases. In a photoconductor, gain is determined by how long a photogenerated carrier can survive in the material compared to how fast it travels across the device.^87^

When the carrier lifetime (τ) exceeds the transit time (τ_1_), a single photogenerated carrier can circulate through the external circuit multiple times before recombining. This repeated contribution effectively amplifies the photocurrent, thereby increasing the photoconductive gain. Since responsivity is defined as the photocurrent generated per unit optical power, an increase in effective charge transport events per absorbed photon directly enhances responsivity. Therefore, mechanisms that prolong carrier lifetime, such as trap-assisted carrier confinement in the Mg-doped inhomogeneous InGaN layer, are proposed as a highly probable pathway to increase photoconductive gain when τ > τ_1_ to a relatively higher photoconductive gain (∼2.01), indicating self-powered photoresponse behavior.^77^ However, at higher applied bias, the stronger electric field improves carrier extraction. It reduces trap-assisted recirculation, leading to a decrease in gain toward unity despite the increased responsivity from more efficient carrier collection.

The photodetector performance of the fabricated Mg-doped InGaN device was compared with previously reported InGaN-based photodetectors employing different device architectures and operating conditions, as summarized in Table 3. Within GaN-based photodetectors, Li et al. demonstrated a CdS/GaN heterojunction device integrated into a Hadamard single-pixel UV imaging system at 365 nm, operating in a self-powered mode with a responsivity of 2.4 mA/W and detectivity of 1.34 × 10^10^ Jones, enabled by the built-in junction field for photocurrent modulation and computational image reconstruction.^91^ In comparison, Mishra et al. reported nonpolar GaN nanostructure-based UV photodetectors at 325 nm under 3 V bias, achieving higher responsivity (25.9 mA/W) and detectivity (2.83 × 10^8^ Jones) due to enhanced surface-to-volume ratio and surface-state-assisted carrier separation.^92^

Within InGaN-based MSM photodetectors, device performance is strongly governed by both composition control and interface engineering. Surender et al. reported composition-controlled In_*x*_Ga_1–*x*_N MSM photodetectors grown by MOCVD, where optimized In_0.20_Ga_0.80_N exhibits a responsivity of 112 mA/W at 360 nm under 0.5 V bias, with a detectivity of 6.32 × 10^10^ Jones.^26^ The improved performance is attributed to increased indium incorporation, which enhances UV absorption via bandgap narrowing and modifies the defect. In contrast, Wang et al. introduced GaN interlayers into In_0.15_Ga_0.85_N MSM photodetectors via pulsed NH_3_ growth to improve the metal/semiconductor interface quality, achieving a much lower responsivity of 8.5 mA/W at 420 nm under 5 V bias.^93^ Although the interlayer strategy aims to suppress interface recombination and trap-assisted losses, the overall photoresponse remains limited by inefficient carrier transport, which is commonly associated with MSM structures due to limited junction-driven carrier separation and trap-limited transport.

In InGaN/GaN multiple quantum well (MQW) photodetectors, device performance is strongly governed by quantum confinement, strain engineering, and optical-field enhancement strategies. Fan et al. reported a filterless narrowband MQW photodetector with a responsivity of 277 mA/W at 365 nm under 6 V bias, attributed to wavelength-selective absorption and strong carrier confinement within the quantum wells.^94^ Chen et al. demonstrated an Ag nanoparticle-coupled InGaN/GaN MQW photodetector with a responsivity of 25.7 mA/W at 405 nm under 2.5 V bias, where plasmonic nanoparticles enhance local electromagnetic fields and improve light absorption.^95^ In contrast, Tsai et al. introduced a prestrained superlattice interlayer in InGaN/GaN MQW photodiodes, thereby mitigating strain relaxation and improving interface quality, resulting in more stable carrier transport under reverse bias.^96^ This structural engineering enables microsecond-scale response times (4.6/5.0 μs) and supports reliable operation for optical wireless communication applications.

Cu_2_O-based InGaN heterojunction photodetectors exploit the built-in electric field of the p–n junction to achieve self-powered photodetection. Zhang et al. reported a self-powered n-InGaN/p-Cu_2_O microcrystal photoelectrochemical photodetector operating at 405 nm, in which efficient carrier separation at the heterojunction enables visible-light detection without an external bias.^97^ Wang et al. reported a bare InGaN nanowire photoelectrochemical photodetector operating at 532 nm under zero bias, achieving a responsivity of 21.5 μA/W and a detectivity of 3.46 × 10^13^ Jones.^98^ The device operates via the intrinsic photoelectrochemical behavior of InGaN nanowires, in which the large surface area facilitates electrolyte interaction and enables self-powered carrier separation under visible-light illumination. This work highlights bare InGaN nanowires as a baseline PEC system for visible-light photodetection before heterojunction engineering.^37,106,107^

The key specialty of the graphene/ZnO/Si nanowire heterojunction photodetector reported by Bansal et al.^99^ lies in its synergistic architecture, where bilayer graphene provides a high-mobility carrier extraction pathway, the ZnO/Si heterojunction forms a built-in electric field enabling efficient charge separation under −0.5 V bias, and the Si nanowire array enhances optical absorption via light trapping. This combined effect results in a responsivity of 280 mA/W and a detectivity of 4.2 × 10^10^ Jones at 480 nm. In a related study, Bansal et al. (2025) employed TCAD simulations on a graphene/ZnO nanowire photodetector to isolate the role of the graphene/ZnO interface, confirming that Schottky barrier modulation and drift–diffusion transport dominate the UV photoresponse mechanism.^105^

The photodetector performance of the fabricated Mg-doped InGaN device was compared with previously reported GaN and InGaN-based photodetectors employing different device architectures and operating mechanisms, as summarized in Table 3. The present device exhibits a responsivity of 24 mA W^–1^ and a detectivity of 6.56 × 10^8^ Jones under 6 V bias. These values are within the range reported for InGaN-based photoconductive devices; however, the present measurements were obtained using an indium-dot two-probe configuration without a lithographically defined electrode geometry. Compared with heterojunction photodetectors, such as CdS/GaN and Cu_2_O/InGaN devices, which benefit from built-in electric fields for efficient carrier separation, the present device relies primarily on photoconductive transport within the Mg-doped InGaN layer. Similarly, InGaN/GaN MQW photodetectors generally exhibit higher responsivity owing to quantum confinement effects, enhanced carrier localization, and engineered heterointerfaces that improve carrier collection efficiency. The responsivity obtained in this work is comparable to several reported GaN and InGaN-based photoconductive devices, despite the absence of heterojunction engineering, plasmonic enhancement, quantum-well confinement, or photoelectrochemical mechanisms. This suggests that Mg-induced compositional inhomogeneity and associated localized states can contribute to carrier confinement and photoconductive gain.

The I-T-dependent measurements were conducted to study the transient response of the samples. A mechanical chopper was utilized to periodically modulate the UV light source, allowing precise time-resolved current–time (I–T) characterization.^108^ The transient photocurrent profiles of the device annealed at 600 °C. The device demonstrated excellent repeatability and stability, with distinct transitions between the illuminated (ON) and dark (OFF) states. The rise time (τ_*r*_) is the time for photocurrent passing from 10% to 90% of its peak value (τ_*r*_ = τ_90%_ – τ_10%_), and the decay time (τ_*d*_) is for that from 90% to 10% (τ_*d*_ = τ_10%_ – τ_90%_) based on Figure 9.^109^ In these definitions, τ_10%_ and τ_90%_ indicate the times at which the photocurrent reaches 10% and 90% of its steady-state response value, respectively.

**Figure 9:**
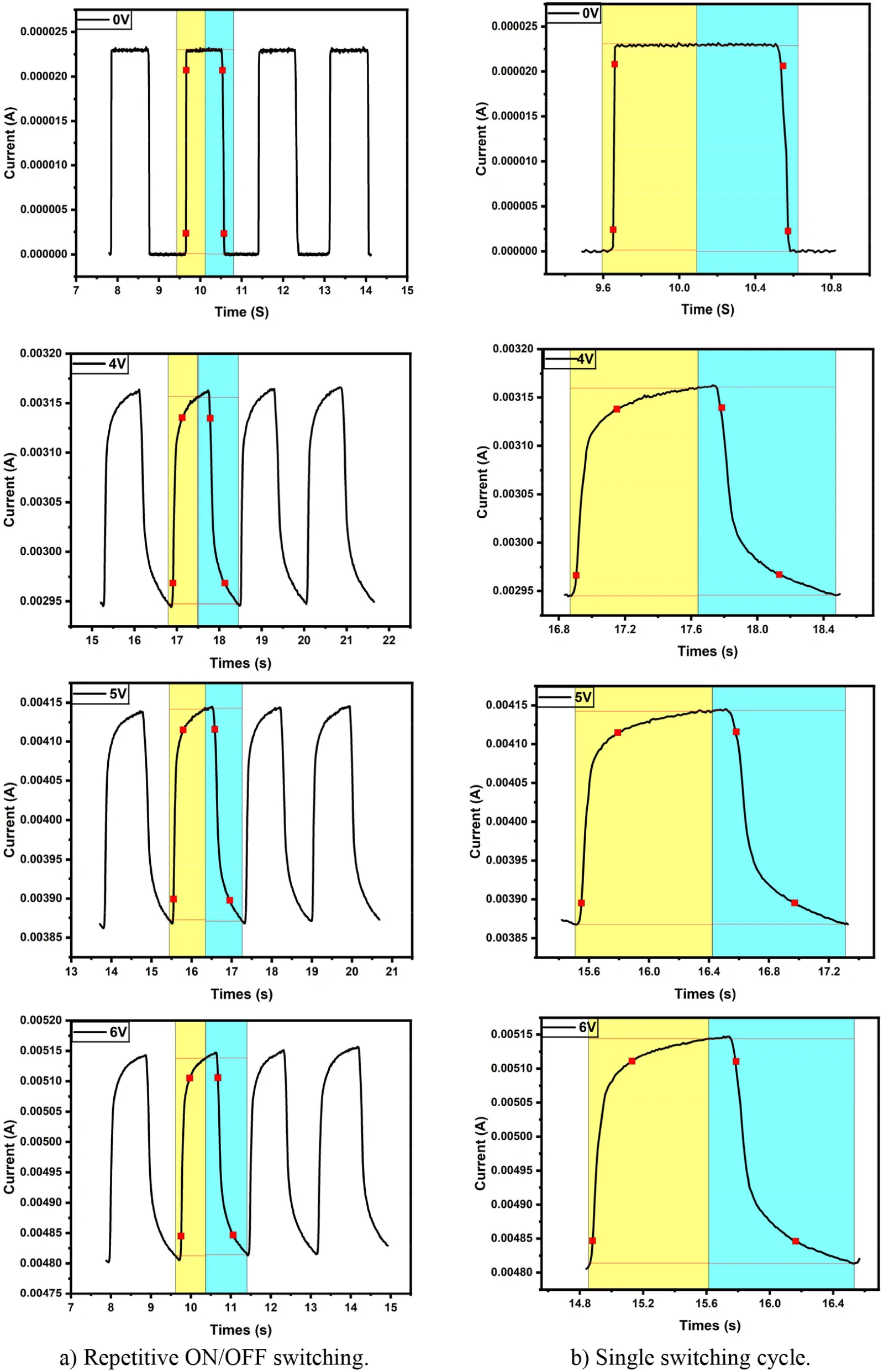
Normalized time-resolved photocurrent behavior of InGaN/GaN photodetector measured at bias voltage of 0, 4, 5, and 6 V and under UV light on/off switching.

The transient response of the Mg-doped InGaN/GaN photodetector under 365 nm UV illumination was investigated to evaluate its dynamic performance. Figure 10 shows the bias-dependent rise time (τ_*r*_) and decay time (τ_*d*_) measured at different applied voltages (0, 4, 5, and 6 V), providing insight into how the applied bias influences the speed of photoresponse in the device. Notably, shorter τ_*r*_ and τ_*d*_ values correspond to faster response speeds, which are critical for achieving high detection capability and reliable operation in UV sensing applications.^110^ The increase in photocurrent amplitude with applied bias reflects enhanced field-assisted carrier transport under a stronger electric field, leading to improved carrier collection and reduced recombination during transit.^64^ At the same time, the fast and repeatable response indicates efficient carrier extraction and reduced trap-assisted recombination,^56^ consistent with the high mobility and low defect density revealed by Hall-effect and structural analyses. The absence of significant current decay or persistent photoconductivity further confirms the effectiveness of the 600 °C annealing process in stabilizing defect states.^111^

**Figure 10:**
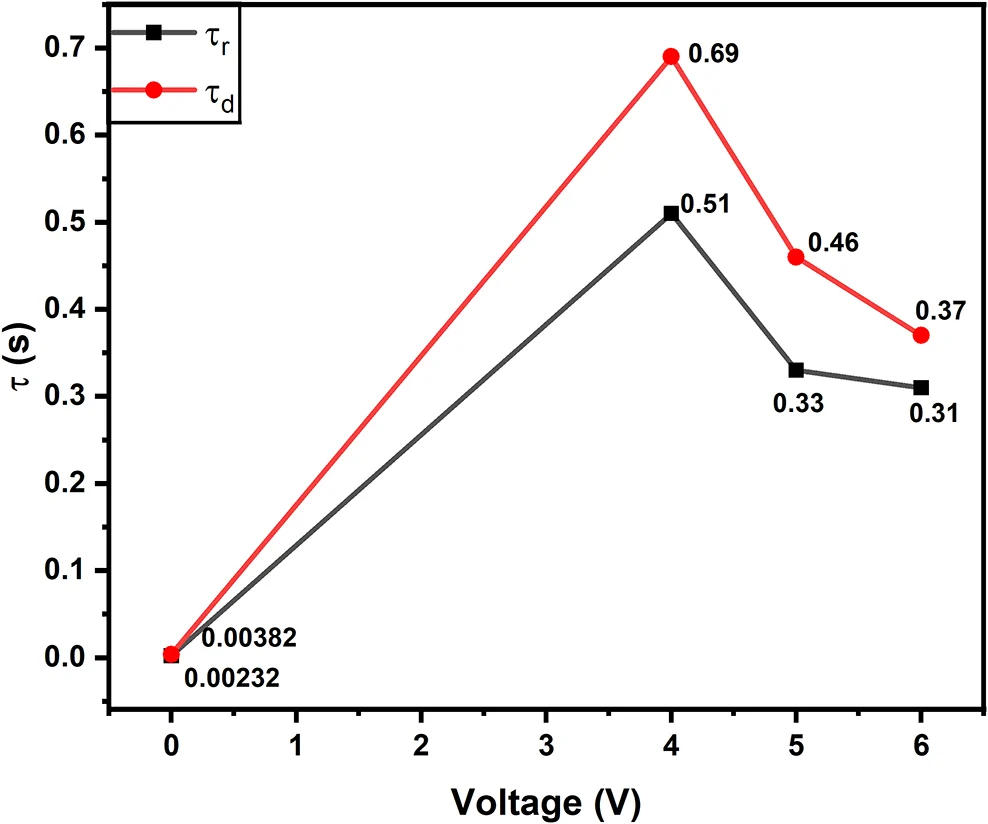
Bias-dependent rise time τ*_r_* and decay time τ*_d_* of under Mg-doped InGaN/GaN photodetector UV illumination of 365 nm and at a bias voltage of 0, 4, 5, and 6 V.

As the bias voltage increases, both τ_*r*_ and τ_*d*_ initially increase, likely due to enhanced carrier trapping and detrapping at defect states and localized potential fluctuations in the InGaN layer. At higher bias (5–6 V), the reduction in both rise and decay times suggests that the stronger electric field enhances carrier transport and extraction, leading to faster carrier collection and a reduced effective recombination probability. Overall, the time-resolved measurements confirm that the Mg-doped InGaN/GaN photodetector exhibits self-powered operation, fast response, and stable performance, with a bias-dependent photoresponse governed by effective carrier transport within the device.

## 3 Conclusion

In this work, the ultraviolet photoresponse of Mg-doped InGaN/GaN heterostructures annealed at 600 °C was systematically investigated to establish correlations between structural, optical, and electrical properties. Reciprocal space mapping revealed partial strain relaxation with an indium composition ranging from 4.11% to 8.77%, while AFM showed step-terrace surface features characteristic of strain relaxation. UV–vis spectroscopy exhibited a steep absorption edge in the UV region, indicating good optical quality and a low density of band-tail states. Taken together, the Hall measurements verify that postgrowth annealing at 600 °C markedly enhances the transport quality of the InGaN layer, yielding an unusual coexistence of high mobility and high carrier concentration. The consequently low resistivity reflects suppressed scattering and efficient carrier conduction, underscoring the excellent electronic quality of the material. These results position the present InGaN layer as a highly promising platform for the realization of high-efficiency optoelectronic and photonic devices. Notably, despite the formation of ohmic contacts, the devices demonstrated a self-powered UV photoresponse, evidenced by stable photocurrent generation at zero external bias. This behavior is attributed to built-in electric fields originating from polarization effects and band offsets at the InGaN/GaN heterointerface. At the same time, the ohmic contacts facilitate low-resistance carrier collection without suppressing the internal electric fields responsible for self-powered operation. Overall, the results highlight the critical roles of strain relaxation, surface morphology, optical quality, and internal electric fields in governing UV PD behavior. Although p-type conductivity was not achieved, the observed steep absorption edge and self-powered UV photoresponse demonstrate promising potential for Mg-doped InGaN-based UV photodetectors. Future studies may focus on enhanced Mg-activation strategies and device optimization to further improve performance.

## 4 Methodology

In this work, Mg-doped InGaN/GaN heterostructures were grown on 2-in. c-plane sapphire (Al_2_O_3_) substrates by metal–organic chemical vapor deposition using an SR4000-HT reactor (Taiyo Nippon Sanso). Trimethylgallium (TMGa), triethylgallium (TEGa), and trimethylindium (TMIn) were used as group-III precursors, with ammonia (NH_3_) as the nitrogen source. Following thermal cleaning at 1100 °C, a 20 nm low-temperature GaN buffer layer was deposited at 570 °C, followed by a 3.2 μm undoped GaN layer grown at 1120 °C. The InGaN layer was grown at 760 °C using TEGa and TMIn flow rates of 17.535 and 46.998 μmol min^–1^, respectively, corresponding to a TMIn/(TMIn + TEGa) ratio of 72.8%, while NH_3_ was supplied at 24 SLM (V/III = 16,562). Hydrogen and nitrogen were used as carrier gases for GaN and InGaN growth, respectively. Magnesium doping was introduced using bis­(cyclopentadienyl)magnesium (Cp_2_Mg) at a molar flow rate of 0.397 μmol min^–1^, yielding an estimated Mg concentration of 1.35 × 10^20^ cm^–3^. A 100 nm-thick InGaN layer was obtained with a growth time of 50.5 min. The samples were postannealed in an ambient at 600 °C for 15 min.

The as-grown wafer was cleaved into approximately 10 × 10 mm^2^ samples for structural, morphological, optical, and electrical characterization. High-resolution X-ray diffraction (HRXRD) reciprocal space mapping (RSM) of the asymmetric GaN (105) reflection was performed using a Bruker D8 Discover system to determine the in-plane and out-of-plane lattice parameters of the InGaN layer. The strain state and degree of relaxation were quantitatively evaluated using LEPTOS software by fitting the experimental RSM data. Surface morphology was examined by atomic force microscopy (AFM; Bruker Dimension Edge) in tapping mode over a 5 μm × 5 μm scan area at a scan rate of 0.4 Hz, from which the root-mean-square (RMS) roughness and surface topography were analyzed. Optical properties were investigated using Carry 5000 NIR UV–Vis spectroscopy, and the optical bandgap was determined from the Tauc plot by extrapolating the linear region of the (*αhν*)^2^ versus photon energy (*hν*) curve.

To form electrical contacts, indium dots were deposited along the edge of the InGaN layer, as shown in Figure 11, serving as ohmic contacts. The active area of the sample was approximately 1 cm^2^. Electrical properties were characterized at room temperature using Hall-effect measurements with an HMS-5000 system in the Van der Pauw configuration. The carrier concentration, Hall mobility, and resistivity were extracted from the measured Hall voltage. All measurements were conducted at a fixed excitation current in the microampere (μA) range to ensure stable, reliable electrical characterization.

**Figure 11:**
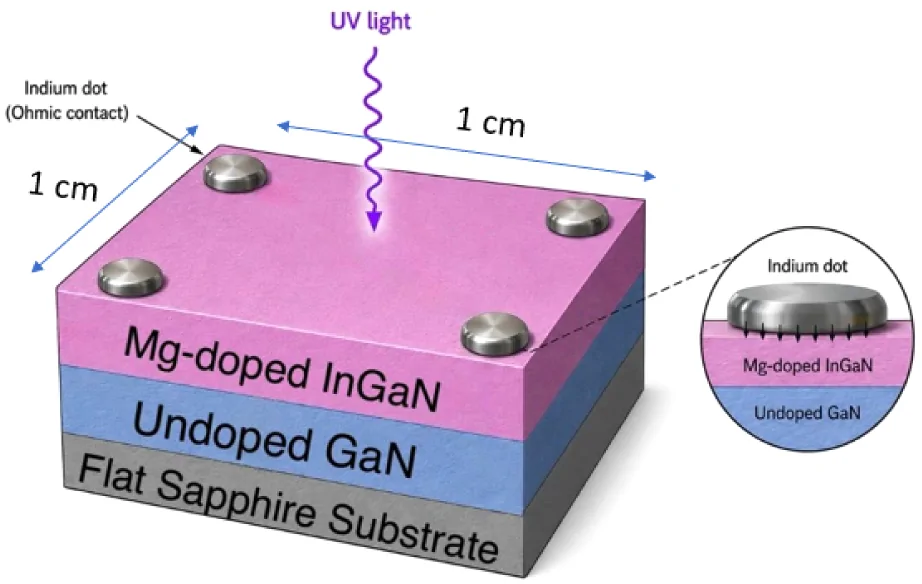
Schematic diagram of the Mg-doped InGaN/GaN heterostructure on a flat sapphire substrate with four indium (in) contact dots.

The photoresponse of the samples was characterized using a custom-built measurement setup, as shown in Figure 12. The device was connected to a two-point probe system with a Keithley source meter, with electrical contact established using crocodile clips attached to the electrodes as shown in Figure 12b. The InGaN was characterized using a two-point probe at two adjacent corners of indium dot contacts to evaluate its photoresponse properties. The indium dots were used as simple probing electrodes on the InGaN surface to investigate lateral carrier transport and photoresponse behavior. Due to the indium-dot contact geometry, the current path follows a nonideal point-contact configuration, where the effective contact area may vary slightly depending on contact spreading at the metal/InGaN interface, with a channel distance of approximately 0.8 cm defining the carrier transport path. Consequently, the measured resistance includes contributions from both the InGaN layer and the indium/InGaN contact interfaces. The contact resistance of approximately 0.2 kΩ per indium contact indicates low resistance and the effective resistance extracted from the linear region of the dark I–V curves was on the order of 10–12 kΩ. Therefore, the extracted electrical parameters should be interpreted as effective device-level values rather than intrinsic material properties. Despite these limitations, the configuration is suitable for comparative evaluation of the photoresponse behavior under different bias conditions

**Figure 12:**
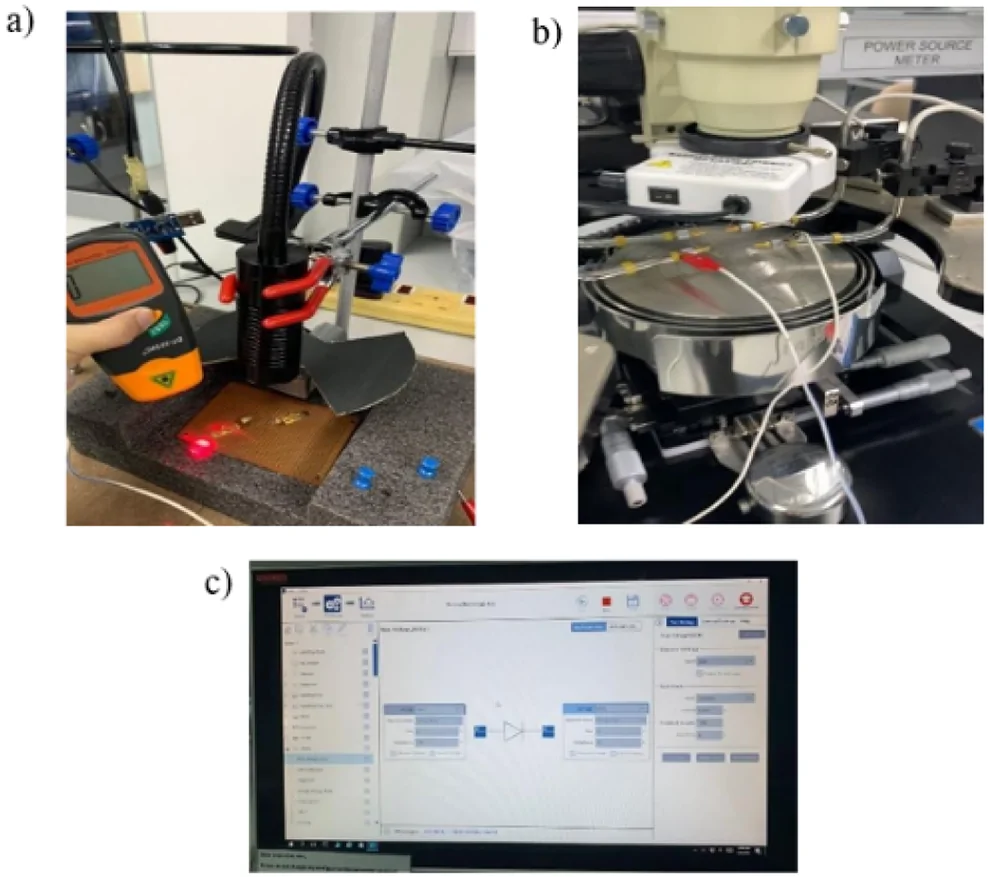
Pictures represent: (a) the light source and the optical chopper, (b) the setup of PDs connected with the two terminals of a probe station system, and (c) the schematic diagram of the current–voltage (I–V) measurements using the keithley source meter.

The devices were illuminated with UV light at 365 nm and 20 mW cm^–2^. The distance between the light source and the sample surface is 6 cm. A rotating chopper periodically modulated the light exposure at approximately 60 m s^–1^. Current–voltage (I–V) characteristics were measured over a bias range of −6 to +6 V under both dark and UV-illuminated conditions to evaluate the device’s electrical behavior. The performances of the devices such as responsivity (*R*), photoconductive gain (*G*), noise-equivalent power (*NEP*), and specific detectivity (*D* ^*^) were calculated using standard photodetector relations based on the measured photocurrent and dark current, with an assumed bandwidth of 1 Hz. In estimating *D* ^*^, the noise was assumed to be dominated by dark leakage current.

## Data Availability

Data will be made available on request.
